# Editorial: Difficult and Severe Asthma in Children

**DOI:** 10.3389/fped.2019.00205

**Published:** 2019-05-17

**Authors:** Andrew Bush, Renato Cutrera, Giorgio Piacentini, Francesca Santamaria, Nicola Ullmann

**Affiliations:** ^1^Professor of Paediatrics and Head of Section (Paediatrics), Imperial College London, London, United Kingdom; ^2^Professor of Paediatric Respirology, National Heart and Lung Institute, London, United Kingdom; ^3^Consultant Paediatric Chest Physician, Royal Brompton Harefield NHS Foundation Trust, London, United Kingdom; ^4^Pediatric Pulmonology and Respiratory Intermediate Care Unit, Sleep and Long-Term Ventilation Unit, Academic Department of Pediatrics, Bambino Gesù Children's Hospital, Rome, Italy; ^5^Professor of Paediatrics and Head of Paediatric Section AOUI Verona, University of Verona, Verona, Italy; ^6^Department of Translational Medical Sciences, Pediatric Pulmonology, Federico II University, Naples, Italy

**Keywords:** allergy, atopy, endotype, phenotype, eosinphil, airway inflammation, airway obstruction

The global burden of asthme and its treatment is huge (1), and is reviewed by Ferrante and La Grutta. Part of the problem requires a political solution; in large parts of the world, children do not have access to basic asthma treatments (2) [and shamefully, this is not just a low and middle income setting problem; children in the so-called developed world die of asthma because their parents cannot afford to pay for treatment (3), and there is no uniform health coverage].

Of course, asthma is an umbrella term covering a multiplicity of airways diseases in children (4, 5), many of which differ from adult disease (6, 7). Oksel et al. cover the topic of phenotype discovery and its clinical relevance. They explore the complimentary approaches of unsupervised discovery and investigator-imposed approaches. They stress the need for cross-disciplinary approaches if we are to maximize the opportunities offered by big data. Licari et al. also underline the heterogeneity of the disease and highlight the difficulties of measuring control and risk, especially in severe asthma. Importantly, their focus is on factors which can be modified (8).

There can be few greater challenges than the child with asthma who does not respond to treatment as anticipated, and this is the main theme of this Topic. The human misery of chronic symptoms and the ever-present risks of acute attacks including death^1^, impaired lung growth with the likely consequence of chronic obstructive pumonary disease (9), and side-effects of medications, especially systemic corticosteroids, are a huge burden to a thankfully small, but certainly significant, group of children.

## Getting Basic Management Right

This is clearly essential, because if this is done, many children with apparently severe asthma turn out to respond to the usual basic strategies (10–12).

### Is It Asthma, and Is It Only Asthma?

Ullmann et al. tackle this topic, and make the important point that pediatricians consider themselves able to diagnose asthma clinically, and all too often commit the child to long term treatment without making a single objective measurement! It is impossible to think of another chronic condition in which simple tests can be made to support the diagnosis, but are omitted (13). The result, that a diagnosis of asthma is frequently wrong, is all too predictable. They also highlight “asthma plus,” co-morbidities which may mimic or worsen asthma, and may lead to inappropriate escalation of treatment. As discussed, the differential diagnosis of asthma in children is wide; and inhaled foreign body is rarely helped by even the highest dose of inhaled corticosteroids (ICS)!

### Getting a Measurement Culture Established

Twenty-first century asthma management *must* be based on a measurement culture. Two important reviews are measurement of airway obstruction by Calogero et al. and assessment of airway inflammation by Tenero et al. Calogero et al. review the role of spirometry and forced oscillation in the assessment of children with asthma. The bedrock of physiology is spirometry, including the acute response to a short-acting β-2 agonist. Of course there is no “gold standard” physiological test, but the more tests that are done which fail to document variable airflow obstruction (including home lung function monitoring and exercise challenge testing) the more carefully another diagnosis should be sought. Persistent airflow limitation will almost certainly track throughout life, and the child with severe asthma is the father of the man with chronic obstructive pulmonary disease. We also need to look beyond spirometry to more sophisticated physiological testing to understand really severe asthma. Treatment of eosinophilic airway inflammation is with ICS, but so often we fail to measure what we are treating; would a cardiologist treat hypertension without measuring blood pressure? Furthermore, there is increasing evidence that untreated Type 2 inflammation even in apparently well controlled asthmatics is a marker of risk for an acute asthma attack (14). There is most evidence on exhaled nitric oxide and induced sputum and peripheral blood eosinophil count, but Tenero et al. have rightly gone beyond these measures to discuss many other potential inflammometry tools. If we are to move from phenotypes to endotypes, much more specific markers using the techniques they discuss will be very important.

### Getting the Basics Right

It is clear that guidelines on their own, without consideration of implementation strategies, are insufficient to deliver improved outcomes, but paying heed to them is really important. Tesse et al. review both pharmacological and, equally importantly, non-pharmacological guideline recommendations; following these is an essential pre-requisite before diagnosing severe, therapy resistant asthma. However, the best medications in the world are useless unless they are taken, and poor adherence to treatment is difficult to detect and even more difficult to correct. Boutopoulou et al. carried out a systematic review of the various strategies to improve adherence. There was a great diversity of methods, testifying to how frustrating a problem this is to deal with. Encouragingly, in general all of the interventions improved adherence to some extent, but none was perfect, and the authors clearly identified a need for more research. Certainly the experience of most pediatric pulmonologists is that, if adherence could be got right, a lot of apparently therapy resistant asthma would disappear. The commonest cause of resistance to asthma therapy is that the therapy is gathering dust on the shelf.

### Getting the Basic Treatment Right

There is of course more to treatment than just reaching for more medications; treatment of severe asthma includes attention to social and environmental factors, and co-morbidities (15). Early multiple aeroallergen sensitization is associated with progression to preschool wheeze (16), but in established allergic asthma, the combination of aeroallergen sensitization and exposure, and viral infection are strongly associated with risk of a severe attack (17). The relationship between allergy and severe asthma is reviewed by Arasi et al., who also discuss how other risk factors such as smoking play into the pathophysiology of severe asthma, and rightly stress the need for more research.

## What are the Pathophysiological Mechanisms?

### Phenotypes and Endotypes

This subject was reviewed by Bush. Currently, all too many patients are treated without any measurements being made. The current approach should be to phenotype the airway, looking for treatable traits such as eosinophilic airway inflammation. However, we cannot assume that airway eosinophilia is synonymous with Type 2 inflammation (18, 19), and we need to move to determining endotypes, specific pathobiological pathways, especially as a multiplicity of new biologicals will become available.

### New Kid on the Block: Macrophage Biology?

Alveolar macrophages are a key player in host defense, but have been little studied in asthma. Kulkarni et al. used induced sputum at sea level and then in a low allergen environment at altitude. They show that allergen or possibly lipopolysaccharide may drive enhanced phagocytosis by asthmatic alveolar macrophages. More work is needed to see how this fits into the Type 2 inflammation paradigm, which was unchanged.

## Beyond Guidelines: Innovative Therapies in Severe Asthma

### The Dawning of the Age of Biological

After years of just having “the blue and brown inhaler” or variants thereof, we are now in a new age of in particular novel biologicals, reviewed by Maglione et al. However, they rightly highlight the vital importance of getting safety and efficacy data in children; we cannot assume that severe asthma is the same across the whole life course. If the biologicals deliver on their promise, then oral corticosteroids as long term therapy in children may become consigned to the history books.

### Immunotherapy?

Corticosteroids are potent improvers of symptoms, whether given orally or by inhalation, but multiple studies have shown they are palliative, not curative of asthma (20–22). The only truly disease-modifying approach is allergen immunotherapy (23). Tosca et al. review the roles of immunotherapy in preventing the development of asthma (surely our ultimate aim) and also in established disease, which may be a more risky approach.

### From Left Field: Herbal Medications

Probably few if any readers know much about the herbal medication *Astrologus*. In an intriguing study, Wang et al. treated children for 6 months with this medication and showed an increase in spirometry and quality of life; they also showed impressive effects on regulatory T-cells, proposing very plausible mechanistic biological pathways. This herbal approach is not yet ready for prime time in the West, but is an important reminder of how much we still have to learn from Eastern practice.

## Summary and Conclusions

This Topic offers diverse perspectives on really severe asthma in children, especially focusing on the treatable. The 60 distinguished authors have brought a unique collective wisdom for which we are grateful. The Editors have certainly learned a lot as the Topic has evolved, and we commend it to future readers; the numbers who have already gone to these articles testify to the great job the authors have done.

## Author Contributions

AB wrote the initial draft. All authors reviewed and approved the manuscript.

### Conflict of Interest Statement

The authors declare that the research was conducted in the absence of any commercial or financial relationships that could be construed as a potential conflict of interest.

## Footnotes

^1^https://www.rcplondon.ac.uk/projects/national-review-asthma-deaths
